# Advancing cadmium bioremediation: future directions for CadR display strategies

**DOI:** 10.3389/fbioe.2025.1720570

**Published:** 2025-11-18

**Authors:** Chang-ye Hui

**Affiliations:** Shenzhen Prevention and Treatment Center for Occupational Diseases, Shenzhen, China

**Keywords:** cadmium bioremediation, CadR surface display, biosorption, bioaccumulation, environmental microbes

## 1 Introduction

Cadmium (Cd) is a highly toxic heavy metal that poses significant environmental and health risks through its accumulation in natural resources and subsequent transfer along the food chain. Cd contamination in soil, water, and air has become a global concern due to its persistence and non-biodegradability, leading to severe ecological and human health impacts. Traditional remediation methods, such as chemical reduction (Hashim et al., 2011) and electro-remediation (Li et al., 2025), are often costly and can introduce additional environmental burdens. In contrast, bioremediation, which leverages the natural ability of microorganisms and plants to accumulate and sequester heavy metals, offers a more sustainable and eco-friendlier alternative (Kumar et al., 2021). This approach not only mitigates Cd toxicity but also enhances the resilience of ecosystems. Recent environmental biotechnology advancements have expanded the potential of bioremediation strategies, including Cd(II) responsive metalloregulator CadR-displayed surface-engineered organisms (Hui et al., 2021a). This opinion article aims to review the current state of CadR-displayed technologies, highlight their limitations, and propose future directions for enhancing their efficiency and applicability in real-world scenarios.

## 2 Environmental microbial resistance mechanisms against Cd(II)

Microorganisms, particularly bacteria, have evolved various resistance mechanisms to survive in Cd(II)-polluted environments. These mechanisms include biosorption, bioaccumulation, efflux systems, and the expression of metallothioneins and other detoxifying proteins (Hansda et al., 2016). Biosorption is a rapid, metabolism-independent process where Cd(II) ions are captured by functional groups on the bacterial cell surface, such as carboxyl, phosphate, and hydroxyl groups (Hui et al., 2021a). Bioaccumulation involves the active uptake of Cd(II) ions into the bacterial cytoplasm, often facilitated by specific transporters. Efflux systems actively pump Cd(II) ions out of the cell to reduce intracellular concentrations. Metallothioneins and other detoxifying proteins bind Cd(II) ions, sequestering them and reducing their toxicity (Hu et al., 2025). These mechanisms are regulated by metal-responsive transcription factors, such as CadR in *Pseudomonas putida* and ZntR in *Escherichia coli*, which activate the expression of resistance genes in response to Cd(II) exposure (Jung and Lee, 2019).

CadR (Cadmium Resistance Regulator) is a key transcriptional regulator from the MerR family, primarily found in bacteria such as *Pseudomonas putida* and *Staphylococcus aureus* (Lee et al., 2001). It functions to regulate the expression of cadmium resistance operons, such as *cad* operons, acting as a repressor in the absence of Cd(II) and as an activator when Cd(II) is present (Barrows et al., 2025). Structurally, CadR consists of DNA-binding, metal-binding, and dimerization domains, which facilitate its regulatory role (Guo et al., 2024a). The metal-binding domain specifically recognizes Cd(II) ions, triggering a conformational change that activates downstream resistance genes (Liu et al., 2019). Given the robustness and specificity of the *cad* operon, it has become an ideal template for developing whole-cell biosensors and bioadsorbents for Cd(II) detection and removal. The *cad* operon, which includes genes for Cd(II) transporters and regulatory proteins, provides a strong foundation for engineering bacteria with enhanced Cd(II) resistance and remediation capabilities. Recent studies have demonstrated that genetically engineered bacteria can significantly enhance Cd(II) removal efficiency through the surface display of Cd(II)-binding proteins and peptides, as well as by optimizing intracellular sequestration pathways (Hui et al., 2021a).

## 3 CadR surface display: current advances and limitations

The surface display of CadR on microorganisms has garnered significant interest due to its potential to enhance the adsorption of Cd(II) ions. Recent studies on the surface display of CadR are summarized in Table 1. All of these studies are currently at the proof-of-concept stage and have not yet been tested with actual contaminated environmental samples. Therefore, the specific removal efficiencies still need further evaluation. Below, we offer a detailed analysis of these studies.

**TABLE 1:** Surface-display of CadR for Cd^2+^ ions biosorption.

Strain	Surface anchoring motif	Metalloregulator	Specificity	References
*Pseudomonas aeruginosa*	INP	CadR	Cd^2+^	Tang et al. (2018)
*Escherichia coli*	pGSA	CadR, PbrR	Cd^2+^, Pb^2+^	Han et al. (2025)
*Escherichia coli*	Lpp-OmpA	Truncated CadR	Cd^2+^	Guo et al. (2021)
*Rhodopseudomonas palustris*	OmpA	CadR	Cd^2+^, Pb^2+^	Wang et al. (2025)

One notable study is the chromosomal expression of CadR on *Pseudomonas aeruginosa* for removing Cd(II) from aqueous solutions (Tang et al., 2018). This study demonstrated the successful integration of the *cadR* gene into the chromosome of *Pseudomonas aeruginosa*, resulting in stable expression and high adsorption capacity for Cd(II). The engineered *Pseudomonas aeruginosa* exhibited an adsorption capacity of up to 131.9 μmol/g of Cd(II), highlighting the effectiveness of chromosomal integration for long-term stability and functionality. This approach enhanced the selectivity of the bacteria for Cd(II) and provided a robust platform for potential environmental applications.

Another innovative study is the rational design of a dual-bacterial system for the synchronous removal of antibiotics and Pb(II)/Cd(II) from water (Han et al., 2025). This research introduced a synergistic approach where two different bacterial strains were engineered to target multiple pollutants simultaneously. One strain was designed to degrade antibiotics, while the other was optimized for heavy metal removal, including Cd(II). This dual-system approach demonstrated the potential for addressing complex environmental contamination scenarios by combining the strengths of different engineered bacteria.

Numerous studies have examined the surface display of CadR and its Cd(II)-binding domain (CdBD) on various microorganisms, demonstrating the potential of engineered bacteria for Cd(II) adsorption (Guo et al., 2021). The surface display of CadR has been a common strategy, leveraging the specific binding affinity of CadR for Cd(II) to enhance the adsorption capacity of the host bacteria.

However, a critical question remains unanswered: Can CadR regulators form dimers when displayed on the bacterial outer membrane, as their dimeric state is essential for effective metal ion binding? While molecular dynamics simulations suggest the formation of dimers, direct experimental evidence, such as from crystallography or other structural biology techniques, is still lacking.

A recent study presents another novel approach to Cd (II) bioremediation by engineering the photosynthetic bacterium *Rhodopseudomonas palustris* CGA009 to display the CadR protein on its surface (Wang et al., 2025). This research builds on previous work by integrating the CadR protein into a photosynthetic bacterium, which offers unique advantages such as metabolic versatility and environmental adaptability. The engineered *Rhodopseudomonas palustris* CGA009 significantly improved Cd(II) adsorption, with a maximum removal rate reaching 95.6%. The study also highlighted the potential for using light intensity to regulate the expression of the CadR protein, further optimizing the bioremediation process.

This study, similar to earlier ones, has its limitations. The research focused on the adsorption of four metal ions: Cu(II), Zn(II), Cd(II), and Pb(II). Notably, non-specific Pb(II) adsorption was observed, indicating a need for further optimization to enhance specificity. The study employed optimization techniques, including linker peptides and promoters, to improve surface display efficiency. While these methods showed promise, further optimization is required to enhance the overall adsorption performance.

## 4 Future directions for CadR display strategies

Despite the significant progress made in CadR surface display technologies, several critical gaps remain that limit their widespread application in real-world bioremediation efforts. Previous studies have successfully demonstrated the potential of CadR in enhancing Cd(II) adsorption. However, the specificity and efficiency of these systems still need substantial improvement. Moreover, the majority of current research has focused on model organisms, which may not be suitable for complex environmental conditions. This article aims to address these gaps by proposing innovative strategies to enhance CadR surface display efficiency, specificity, and adaptability to diverse environmental settings.

### 4.1 Exploring CadR homologs for enhanced specificity

A previous study highlights a crucial insight into the variable response specificity of sensors developed using different CadR homologs (He et al., 2021). As is well-known, the binding of dimeric CadR to metal ions activates the expression of reporter genes downstream of the *cad* promoter, which is the molecular mechanism underlying sensing specificity. The specific binding of dimeric CadR to metal ions may be the mechanism by which CadR on the cell surface captures and adsorbs Cd(II). After all, no crystal structure of CadR displayed on the bacterial outermembrane has been elucidated. It suggests that different CadR homologs displayed on the cell surface will vary in their specificity and capacity for capturing and enriching Cd(II).

Protein engineering has been a conventional approach to enhance the specificity of metalloregulators for metal ion binding. Numerous studies have focused on directed mutagenesis of metalloregulators to improve the specificity of biosensors (Cai et al., 2022; Guo et al., 2024b; Shen et al., 2025). However, this approach often overlooks the naturally evolved homologs repository. Most current research is based on CadR from *Pseudomonas putida*, which has notable drawbacks: biosensors developed using this CadR often exhibit cross-reactive nonspecific responses to multiple metal ions, especially Pb(II) and Hg(II) (Hui et al., 2021b; Hui et al., 2022a; Hui et al., 2022b; Zhang et al., 2024), and biosorbents based on this CadR also lack sufficient specificity in adsorption (Wang et al., 2025).

The previous study emphasizes the importance of screening CadR homologs. In the future, we can leverage natural gene repositories, such as those in GenBank, to conduct bioinformatics analyses of CadR homologs. This could lead to the identification of homologs with stronger targeting and higher adsorption capacity for Cd(II). We can enhance the targeting and capacity for Cd(II) adsorption by employing these homologs in surface engineering.

### 4.2 Optimizing anchor proteins and linker peptides

**FIGURE 1:**
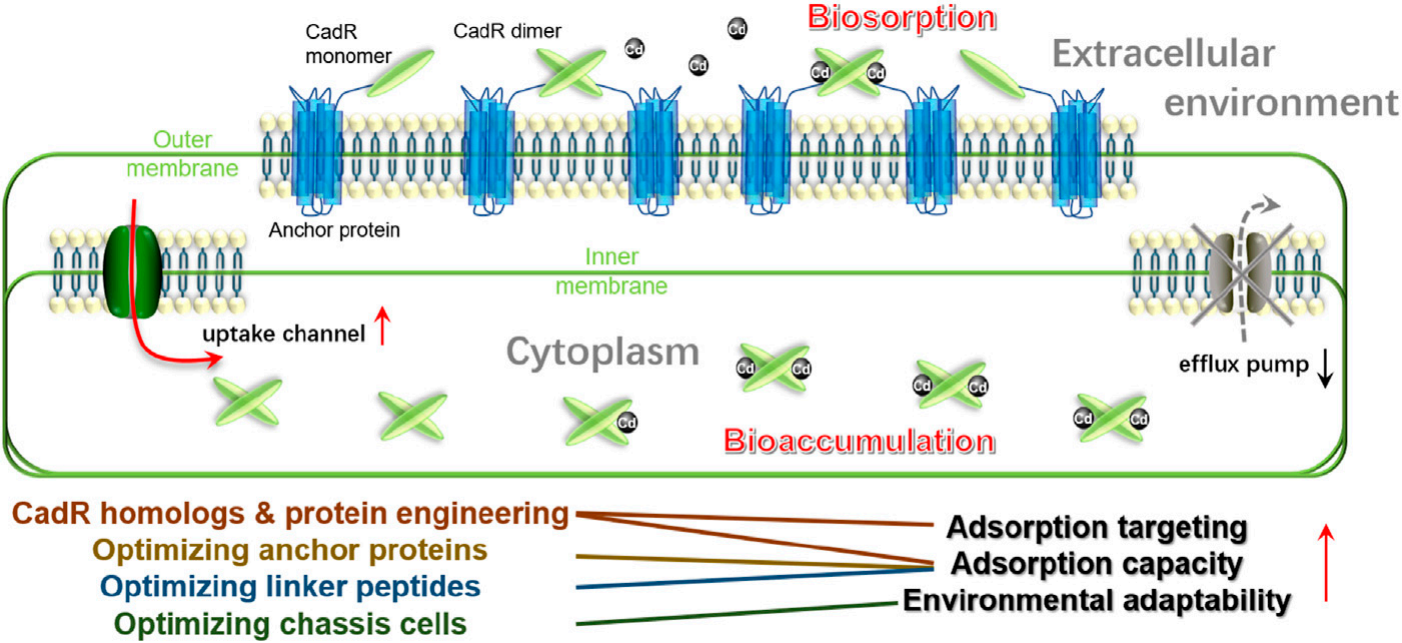
Optimization strategies for CadR-based whole-cell biosorption and bioaccumulation of Cd(II). CadR is displayed on the microbial outer membrane via an anchor protein, forming dimers that capture Cd(II) ions. Future research can focus on screening CadR homologs from natural gene repositories to enhance Cd(II) targeting and adsorption capacity. Additionally, optimizing anchor proteins and linker peptides can improve display efficiency and Cd(II) adsorption. Another focus is combining extracellular biosorption with intracellular bioaccumulation by expressing Cd(II) transporters and sequestering proteins within the cell. Expanding host organisms to environmental microbes and microalgae can enhance the adaptability of surface-engineered cells for environmental remediation.

Our previous study highlighted the importance of optimizing anchor proteins to ensure the target proteins’ successful display and high display efficiency (Guo et al., 2025). Another study further emphasized the significance of selecting appropriate linker peptides, particularly flexible linkers, for the conformational formation of metalloregulator PbrR after surface display (Jia et al., 2020). MerR-like metallloregulators typically bind to their corresponding divalent metal ions in a dimeric form (Jung and Lee, 2019). As shown in Figure 1, we hypothesize that the optimal linker peptide can create minimal spatial hindrance, facilitating the correct formation of dimeric metallloregulators and their subsequent binding to the corresponding divalent metal ions. These strategies have not yet been applied to the display of CadR, and thus, they present promising directions for future research.

### 4.3 Integrating extracellular biosorption with intracellular bioaccumulation

Intracellular sequestration is a resistance mechanism employed by some heavy metal ions, such as Pb(II), to mitigate toxicity (Hui et al., 2023). This strategy can be adapted to enhance the bioremediation of Cd(II) by integrating extracellular biosorption with intracellular bioaccumulation. Specifically, the extracellular biosorption based on CadR can be combined with intracellular bioaccumulation, leveraging CadR to create a synergistic system (Figure 1).

Natural bacteria have evolved various mechanisms for cadmium biosorption and bioaccumulation. Biosorption is a rapid, metabolism-independent process where Cd(II) ions are captured by functional groups on the bacterial cell surface, such as carboxyl, phosphate, and hydroxyl groups (Vijayaraghavan and Yun, 2008). Bioaccumulation, on the other hand, involves the active uptake of Cd(II) ions into the bacterial cytoplasm, often facilitated by specific transporters (Hansda et al., 2016). This process is especially beneficial as it decreases the environmental concentration of Cd(II) and safeguards bacterial cells from its toxic effects. We can achieve this by upregulating the expression of specific membrane transport proteins, such as P-type ATPases and ABC transporters, that facilitate the uptake of Cd(II) into the cell. Knocking out genes that encode efflux pumps, like the CadA protein, prevents the expulsion of Cd(II) from the cell, ensuring that Cd(II) remains inside.

Furthermore, as illustrated in Figure 1, CadR monomers can be expressed inside cells. These monomers dimerize to form a sequestering protein that efficiently sequesters Cd(II) within the cell, thereby minimizing the damage caused by Cd(II) to microbial cells. Such an integrated approach not only enhances the efficiency of Cd(II) removal but also protects the microbial cells from the toxic effects of Cd(II), making the system more robust and sustainable for bioremediation applications.

### 4.4 Expanding host organisms for surface engineering applications

Current research in surface engineering for heavy metal bioremediation, including the display of metalloregulators like CadR, has predominantly focused on model organisms such as *Escherichia coli* (Hui et al., 2021a; Hui et al., 2023; Hui et al., 2024). While these studies have provided valuable insights and demonstrated promising results, applying such engineered bacteria in real-world environments faces challenges due to the complex and variable conditions of natural ecosystems. To address these challenges and enhance the practical applicability of surface engineering strategies, it is essential to expand the range of host organisms from traditional model systems to environmental organisms.

Microalgae, for instance, offer a promising alternative as host organisms for surface engineering. As highlighted in the hypothesis for mercury bioremediation using genetically modified microalgae, these photosynthetic microorganisms possess several advantageous characteristics that make them well-suited for environmental bioremediation (Chokshi et al., 2024). Microalgae have a large surface area-to-volume ratio, which enhances their capacity to contact and adsorb pollutants in water. Additionally, they naturally possess the ability to bind and accumulate heavy metals, making them effective biosorbents.

By leveraging the natural capabilities of microalgae and engineering them to display metalloregulators such as CadR on their surface, we can create a robust system for capturing and removing Cd(II) from contaminated environments. This approach not only capitalizes on the inherent advantages of microalgae but also integrates the specificity and efficiency of CadR in targeting Cd(II). The engineered microalgae can then be deployed in real-world settings, such as polluted water bodies, to remediate Cd(II) pollution effectively.

## 5 Conclusion

In summary, the future directions for CadR display strategies to enhance Cd(II) bioremediation involve multifaceted optimizations. These include exploring diverse CadR homologs to improve specificity and adsorption capacity, optimizing anchor proteins and linker peptides to enhance surface display efficiency, and integrating extracellular biosorption with intracellular bioaccumulation to maximize Cd(II) removal. Additionally, expanding the host organisms to include environmental microbes and microalgae can significantly boost the practical applicability of these strategies. While these advancements hold great potential for real-world applications, challenges such as maintaining microbial viability in complex environments and ensuring long-term stability of the engineered systems must be addressed. Continued research and innovation are essential to overcome these hurdles and realize the full potential of CadR-based bioremediation for Cd(II) pollution.
